# Water Supply1Read at the regular meeting of the Buffalo Sanatory Club, December 14, 1898.

**Published:** 1899-03

**Authors:** Louis H. Knapp

**Affiliations:** Superintendent and Engineer, Buffalo City Water Works


### WATER SUPPLY.[1]

By LOUIS H. KNAPP,
Superintendent and Engineer, Buffalo City Water Works.

IT IS conceded that the selection of the camp has been intelligently and impartially determined upon by the board and the problem now is to supply the camp or camps with a sufficient quantity of pure water for their use and convenience.

*The source of supply.*—If the supply is to be taken from a running stream, the source of the supply should be above all possible contamination, such as fords for crossing the stream and places for watering horses, bathing, and the like. This source should be properly guarded to prevent any possible contamination, for no more important problem connected with the sanitary welfare of the camp can arise than that of the purity of its water supply. Upon the healthfulness of the water depends, in a large measure, the health of its occupants and their good health is one of the most important factors toward a successful campaign.

Conveying the water directly to the camp is solely a matter of convenience and not one of necessity. The important factor is a source of supply that is uncontaminated. This source when found can be almost any distance from the camp. The intake can be located in the stream, if of sufficient depth, or a dam can be constructed so as to impound the water, to make a sufficient depth. Where the soil is of a porous nature a well dug on the bank of the stream, so that the water can filter through the sand and gravel into it, makes a perfect supply. At this point a small pump is placed and the water conveyed to the camp through proper pipes. The pump is small and can be conveniently transported. The supply pipes are of wrought iron with screwed joints, and being light can be conveniently handled and put in place by a detail of men that can be selected from any regiment. The depth of the pipe to be sufficient, say five feet, to protect them from injury from the surface heat of the ground or from frost.

The placing of the pump and laying the pipe to the camp should not require more than twenty-four hours when the supply of water is immediately available. This introduction of water should be followed immediately by the erection of a cypress tank or tanks of sufficient capacity[2] and elevation and at a desirable location to act as a reser-

1. Read at the regular meeting of the Buffalo Sanatory Club, December 14, 1898.

2. The staves of the tanks should be properly marked for convenience of erection and if found advisable a large tree can be conveniently employed for the partial support of the tank.

voir, so that all the taps in the camp will have a good supply if they were all opened at the same time. The water pressure should be about thirty pounds per square inch. If it is less it will result in an unnecessary waste of time in procuring the supply, and if of a greater pressure will result in an unnecessary waste of water.

Next to a clear running stream as a source of a water supply is a spring of sufficient capacity. When a stream or spring is not available for the supply, driven wells can be resorted to, the number and size of the wells depending entirely upon the quantity of water required, the area of the water shed, the water-bearing capacity of the porous material through which the water is drawn or flows.

The force required to operate the pumps wiil generally be found to be steam generated from a portable boiler on wheels, using fuel that is immediately available.

Wind mills have been successfully used in certain localities. The storage tanks then should be greater to provide for the uncertainty of the power at all times.

Hydraulic rams can be used where the fall is sufficient to supply the power to raise the water into the tanks.

Electricity can be profitably used as a motor when the power is available, generally near a large city.

A liberal allowance for all necessary uses about the camp would be about forty gallons per day per capita, or 50,000 gallons per day for each regiment occupying the camp.

The water distribution of a regimental camp should consist of seventeen taps and located as follows: One tap at each company's street, four in each battalion, or twelve in each regiment, and one each at the regimental quartermaster's office, mess, hospital, guard tents and stables. Total, seventeen.

*Dimensions for Pump.*

| | DUPLEX PUMP | | | PIPES | | | |
|---|---|---|---|---|---|---|---|
| | Steam. | Water. | Stroke. | Steam | Exhaust. | Suction. | Supply. |
| Regiment . . . . | 5¼″ | 3½″ | 5″ | ¾″ | 1¼″ | 2½″ | 2″ |
| Brigade . . . . | 8″ | 5″ | 10″ | 1½″ | 2″ | 5″ | 4″ |
| Division . . . . | 10″ | 8½″ | 12″ | 2″ | 2½″ | 7″ | 6″ |

One tank, capacity 50,000 gallons, for each regiment. Supply pipes from four inches to six inches in diameter.

The estimated cost for the source of supply, 1,000 yards from the camp: For regiment, $4,000; for brigade, $6,500; for division, $12,000.

Cost of operating, consisting of fuel oil, attendance and repairs per day: For regiment, $10.00; for brigade, $20.00; for division, $60.00.

